# Latest Insights on Adenovirus Structure and Assembly

**DOI:** 10.3390/v4050847

**Published:** 2012-05-21

**Authors:** Carmen San Martín

**Affiliations:** Department of Macromolecular Structures, Centro Nacional de Biotecnología (CNB-CSIC), Darwin 3, 28049 Madrid, Spain; Email: carmen@cnb.csic.es; Tel.: +34-915855450; Fax: +34-915854506

**Keywords:** adenovirus, structure, maturation, minor coat proteins, core proteins, cryo-electron microscopy, crystallography

## Abstract

Adenovirus (AdV) capsid organization is considerably complex, not only because of its large size (~950 Å) and triangulation number (*pseudo* T = 25), but also because it contains four types of minor proteins in specialized locations modulating the quasi-equivalent icosahedral interactions. Up until 2009, only its major components (hexon, penton, and fiber) had separately been described in atomic detail. Their relationships within the virion, and the location of minor coat proteins, were inferred from combining the known crystal structures with increasingly more detailed cryo-electron microscopy (cryoEM) maps. There was no structural information on assembly intermediates. Later on that year, two reports described the structural differences between the mature and immature adenoviral particle, starting to shed light on the different stages of viral assembly, and giving further insights into the roles of core and minor coat proteins during morphogenesis [1,2]. Finally, in 2010, two papers describing the atomic resolution structure of the complete virion appeared [3,4]. These reports represent a veritable *tour de force* for two structural biology techniques: X-ray crystallography and cryoEM, as this is the largest macromolecular complex solved at high resolution by either of them. In particular, the cryoEM analysis provided an unprecedented clear picture of the complex protein networks shaping the icosahedral shell. Here I review these latest developments in the field of AdV structural studies.

## 1. AdV Structure: A Historical Perspective

Adenoviruses [5] have long been of interest in the basic virology field. They are present in most vertebrates [6]. As experimental systems, they have been extremely useful for investigating many fundamental processes in the eukaryotic cell life, such as splicing and apoptosis. In humans they cause usually mild respiratory, gastrointestinal and eye infections. Although the most common type of AdV infection is subclinical, AdV-induced diseases are responsible for significant morbidity in immunocompromized patients and other susceptible populations [7]. There are at present no approved antiadenoviral drug therapies [8]. Adenoviruses have recently shown promise as vectors for gene transfer into mammalian cells, vaccine delivery and oncolysis [9,10,11,12,13,14,15], although improvements in vector design are still needed [16,17]. Thus, there is currently a three-fold interest on AdV structural studies: first, because of their pathogenicity and the lack of anti-adenoviral drugs; second, because of their potential therapeutic use and the need to improve their efficiency as vectors; third, because of their role as experimental models for complex virus assembly.

**Figure 1:**
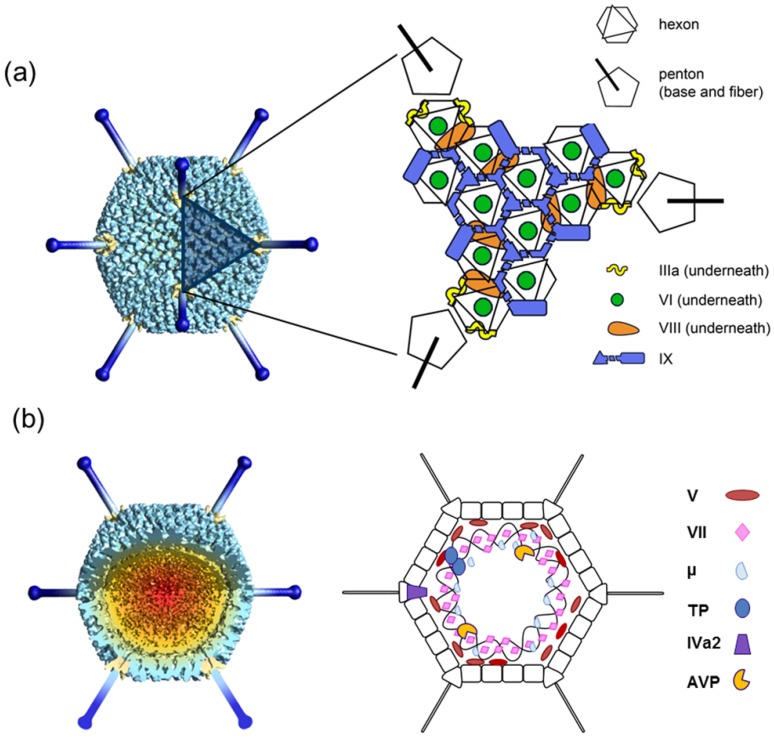
**Overall AdV structure and components.** (**a**) Icosahedral shell organization according to current structural knowledge. The left hand side panel is a model built from a low resolution cryoEM map, with penton bases highlighted in yellow, and fibers built from the crystal structure of the knob and distal shaft [27] in dark blue. The shaded triangle indicates one facet. (**b**) Non-icosahedral components. A segment has been removed from the cryoEM map to show the inner capsid contents. The schematics on the right hand side indicate tentative positions, as little is known about the structure and organization in the virion of the genome and accompanying proteins. Polypeptide IVa2, which binds to the specific packaging sequence in the viral genome, has been reported to occupy a singular vertex in the capsid [28].

Adenoviruses are icosahedral, non-enveloped viruses with a dsDNA genome. Very soon after their discovery [18], it became clear that AdV structural organization was not simple (Figure 1). An impressive amount of experimental work has been performed along this time to converge in our current, detailed knowledge of the AdV capsid organization. In this section I will briefly highlight the main milestones that took us to the situation comprehensively reviewed by W. C. Russell in 2009 [19]. Then, in the rest of the sections I will describe the latest advances in the understanding of adenovirus structure and assembly.

Early electron microscopy (EM) images of AdV allowed counting the number of capsomers forming the capsid [20]. The idea that viruses should be built from identical subunits distributed in highly symmetrical architectures, due to gene economy constraints, had already been proposed [21]. Indeed, these first electron microscopy images showed the AdV capsomers distributed according to the 532 symmetry group, proving that it was an icosahedron. When Caspar and Klug elaborated their theoretical framework for the structure of spherical viruses [22], it was clear that the adenovirus capsid corresponded to a T = 25 triangulation number icosahedral geometry. This predicted that the capsid should have 60 × 25 = 1500 structural subunits: 12 pentamers forming the vertices, plus 240 hexamers. It was assumed that all structural subunits would be chemically identical—but it soon became apparent that the AdV capsid composition did not conform to this assumption.

By combining chromatographic purification with EM of the soluble AdV antigens and of complete virions, Valentine and Pereira [23] (commented by W.C. Russell as a direct witness in [24]) showed that the 6-fold and 5-fold coordinated capsomers were two chemically different entities. According to their neighborhood in the capsid, these were later denominated “hexons” and “pentons” respectively [25]. Valentine and Pereira also produced the first striking images of the extended fibers protruding from each vertex. Later on, the complexity was extended when the virion was shown to contain at least nine different polypeptides [26]. These were named using roman numbers in order of decreasing molecular weight, as revealed by SDS electrophoresis. In this terminology, hexons correspond with polypeptide II, and pentons are composed by polypeptide III (penton base) and IV (fiber).

The next puzzle came from the AdV capsid disruption pattern. Upon mild dissociation conditions, stable substructures composed by nine hexons would reproducibly be generated. These were termed Groups of Nine hexons, or GONs. GONs contain the central hexons in each facet, but not the ones surrounding the penton, or peripentonals [29]. Now these nine hexons, which by definition occupy 6-fold coordinated positions in the T = 25 icosahedron, turned out to be organized in a *p3* net, without any hint of symmetry higher than 3-fold [30]. Further, exhaustive physico-chemical analyses showed that hexons were trimers, and not hexamers, of polypeptide II [31]. Two questions arose from these studies: how could a trimeric protein fulfill the geometrical role of a hexamer? And, what made hexons in the GON different from the peripentonals?

The answer to the first question came from the hexon structure solved by X-ray diffraction. A 6 Å resolution crystallographic model showed that the trimeric capsomer had a pseudo 6-fold hexagonal base, ideally suited to establish a close-packed protein shell for protecting the viral genome [32]. In the opposite side, the trimer had three clearly marked towers that were recognizable in negative staining images of GONs and other subviral structures, and were twisted with respect to the hexagonal base. This facilitated the determination of hexon orientations in the 240 capsomers of the icosahedral facets [33,34]. Chain tracing in the electron density map revealed that the hexagonal shape was achieved by repetition of a structural motif in the base of each hexon monomer: an 8-stranded β-barrel with a “jellyroll” topology [35]. When the hexon homotrimer, rather than the monomer, was considered as the basic building block, it was realized that the icosahedral asymmetric unit (AU) was formed by four independent hexons, placed in four different environments. This is in contrast with the 25 different environments in the AU of a T = 25 icosahedron predicted by Caspar and Klug quasi-equivalence theory. Also, because of the trimeric nature of hexons, the AU is composed by 4 × 3 (hexons) +1 (penton) = 13 independent polypeptides, instead of the predicted 25. It is in this sense that the AdV capsid is often described as *pseudo* T = 25.

The question of why the nine hexon trimers in the GON behaved differently from the peripentonal hexons in disruption studies was solved when it was shown that GONs were formed by two different viral components: hexon, and polypeptide IX [36]. The copy number of IX was determined by ^35^S labeling stoichiometric studies [37]. There are 240 copies of polypeptide IX per virion, with 12 copies per GON. That is, polypeptide IX is associated most intimately with hexons in the GON. The location of polypeptide IX in the GON was directly observed for the first time in difference maps where a GON model constructed from the crystal structure of hexon was subtracted from 2D EM average images of negatively stained GONs [38]. Four trimers of IX were found reinforcing the interactions between hexons at the icosahedral and a set of local 3-fold symmetry axes that are present only within the GON. No similar binding environment appears between the GON and the peripentonal hexons. Thus, the location of IX explained the defined capsid disruption pattern.

A more complete model for the distribution of minor components in the AdV capsid was obtained when the combination of X-ray and EM data was extended to the third dimension. The first 3D image of the whole virion was obtained from alignment and averaging of only 29 individual virion projections from frozen-hydrated samples, and reached a resolution of 35 Å [39]. The atomic model of hexon, filtered to the same resolution, was fitted to the four independent positions in the AU, to obtain a 3D density model for all hexon copies in the particle. This “hexon only” density was subtracted from the cryoEM map, to reveal the molecular envelope of other icosahedrally ordered capsid components [40]. This was a pioneer study, in which it was shown that it was possible to combine 3D data from X-ray diffraction and EM, even if the resolution range of the two techniques was at the time one order of magnitude away, and the physics originating the signal were quite different. Even at the limited resolution attained, the difference maps showed for the first time the shape of the penton complex, and the location of outer and inner densities reinforcing the capsid at specific positions. These were interpreted with the help of the known copy numbers and molecular weights, and assigned to minor coat proteins IIIa, VI, and IX.

At the end of the 20^th^ century and beginning of the 21^st^, methodological and computing advances resulted in a remarkable take off for structural biology techniques. With regard to the AdV structural components, protein crystallography yielded the atomic structures of the fiber knob [41] and shaft [27], as well as penton base, alone and in complex with the N-terminal fiber peptide [42]. These structures were obtained from isolated proteins or domains; to see how they related to each other within the virion context, they were fitted into cryoEM maps moving fast beyond subnanometer resolution: first at 10, then at 9, then at 6 Å resolution [43,44,45]. Because of the higher level of detail available in these maps, it was possible to start interpreting the difference densities in terms of secondary structure elements, particularly at 6 Å resolution, if long helices were predicted. The improved models resulted in reassigned locations for polypeptides IIIa, VI, VIII, and the C-terminal domain of IX.

One of the new assignments for minor coat protein locations concerned a putative 4-helix bundle located between hexon towers at the icosahedron edges. This density was originally thought to correspond to polypeptide IIIa [40], but in the new studies it was attributed to the C-terminal domain of IX [44]. Polypeptide IIIa was now proposed to occupy a position under the vertices inside the virion. Because this reassignment implied a radical change in the accessibility of polypeptide IIIa to ligands such as antibodies or cellular receptors, and may have created confusion in the field, it is worthwhile to summarize here the evidence supporting it. Viruses lacking polypeptide IX also lack all capsid external densities not attributable to hexons or pentons [45,46]. However, for a while it was not clear if this meant that polypeptide IX was the only externally located minor capsid protein, or if the absence of IX produced the loss of polypeptide IIIa. Exogenous peptides or protein domains fused to the C-terminus of polypeptide IX confirmed its location at the facet edges, and indicated an antiparallel arrangement for its C-terminal 4-helix bundle [47,48]. Finally, a peptide mapping study using viruses with N-terminally tagged IIIa confirmed the location of this polypeptide at an internal position below the vertex region [49].

Most of the structural knowledge on AdV comes from studies on the human types 2 (HAdV-2) and 5 (HAdV-5). In the last years, the range of available AdV known structures has expanded to include the fiber knobs of other AdV species infecting humans or animals; knobs bound to their receptors; hexons of simian and avian AdV; and various fiber chimeric constructs (Table 1). These have all been obtained from isolated proteins, outside of the virion context. No structural information on any of the minor coat proteins has been reported using this strategy. It is likely that the minor coat proteins need the virion context to fold properly.

CryoEM has yielded maps of canine AdV, and the *Atadenovirus* ovine AdV [50,51]. Although the different species and genera differ in some of the minor capsid components, the general virion architecture is conserved. This includes particle size, hexon packing, external reinforcement by minor proteins at the 3-fold axes in the GONs, and internal densities beneath the vertices. CryoEM has also contributed to our current understanding on receptor binding and neutralization for HAdV [52,53,54,55,56,57,58,59,60].

**Table 1:** Structures of AdV capsid proteins solved by X-ray crystallography.

Protein	Species 1	Ligands/Modifications	Reference
Hexon	HAdV-2		[61]
	HAdV-5		[61]
	FAdV-1 (CELO)		[62]
	SAdV-25		[63]
Penton base	HAdV-2		[42]
	HAdV-2	N-terminal fiber peptide	[42]
	HAdV-2	Chimera with RGD loop from HAdV-12	[64]
Fiber shaft	HAdV-2		[27]
	HAdV-2	Fused to bacteriophage T4 fibritin trimerization motif	[65]
Fiber knob	HAdV-2		[66]
	HAdV-3		[67]
	HAdV-5		[41]
	HAdV-7		[68]
	HAdV-11		[68]
	HAdV-11	Consensus repeats SCR1-SCR2 of CD46	[69]
	HAdV-11	Consensus repeats SCR1 to 4 of CD46	[70]
	HAdV-12		[71]
	HAdV-12	Domain 1 of CAR	[71]
	HAdV-12	Knob mutants in complex with domain 1 of CAR	[72]
	HAdV-14		[68]
	HAdV-16		[73]
	HAdV-19	Sialyl-lactose	[74]
	HAdV-21		[75]
	HAdV-21	Consensus repeats SCR1-SCR2 of CD46	[75]
	HAdV-35		[76,77]
	HAdV-37		[74]
	HAdV-37	Sialyl-lactose	[74]
	HAdV-37	Domain 1 of CAR	[78]
	HAdV-37	Sialic acid derivatives	[79]
	HAdV-37	GD1a glycan	[80]
	HAdV-37	Trivalent sialic acid inhibitor	[81]
	CAdV-2		[78]
	CAdV-2	Domain 1 of CAR	[78]
	CAdV-2	Sialic acid	[82]
	CAdV-2	Domain 1 of CAR and sialic acid	[82]
Fiber knob (short fiber)	HAdV-41		[83]
Fiber knob (head domain)	PAdV-4		[84]
Fiber knob (galectin domain)	PAdV-4		[84]
Fiber knob (galectin domain)	PAdV-4	Carbohydrates	[84]
Fiber knob (short fiber)	FAdV-1 (CELO)		[85]
Fiber knob (long fiber)	FAdV-1 (CELO)		[86]

A particularly interesting aspect of AdV was revealed by the observation that this virus, which infect vertebrates, has a striking structural similarity to PRD1, a bacteriophage with an internal membrane [87]. Previously, geometrical considerations based on the low resolution structure of AdV hexon had predicted that pseudo-hexagonal trimers could be used to build larger icosahedral capsids [33]. This prediction has now been experimentally confirmed. In the last years, more members of the PRD1-AdV family have been described or predicted, and the lineage now extends from viruses infecting bacteria or archaea, to the large nucleo-cytoplasmic DNA viruses such as Asfarvirus, Iridovirus and the giant Mimivirus [88]. All these viruses are built from the same kind of double jelly roll, pseudo-hexagonal capsomers arranged in different tiling systems, with triangulation numbers ranging between T = 21 and T = 169 (for those actually solved), and reaching up to 972 < T < 1200 for the giant Mimivirus [89]. Intriguingly, even a scaffold protein of the non-icosahedral vaccinia virus folds as a double jelly–roll pseudohexamer, indicating a possible common ancestor with icosahedral dsDNA viruses [90]. Therefore, information obtained on AdV is likely to apply to a large family of highly complex viruses.

## 2. Reaching Atomic Detail in the Complete AdV Virion

The story of AdV structural characterization runs parallel to that of two structural biology techniques: X-ray crystallography, and electron microscopy. AdV was one of the first viruses to be imaged at the electron microscope [20]; hexon was the first animal virus protein to be crystallized [91], and the longest polypeptide solved by X-ray diffraction in its time [35]. AdV was one of the first experimental systems to benefit from the combination of both imaging techniques, at a time when it was not clear how meaningful such a combination would be. From the first interpretations of negative staining EM micrographs using a low resolution crystallographic model of hexon [92], to the difference imaging between GON EM images and hexon projections [38]; to the sophisticated quasi-atomic models and difference maps derived from three-dimensional cryoEM [40,43,44,45], both techniques were successfully put to the test to keep unveiling new details on this complex virus. The story converged when, in a parallel *tour de force*, the complete virion was solved at atomic resolution both by X-ray diffraction and by cryoEM [3,4].

AdV is the largest complex solved by protein crystallography, and the largest, and one of the few so far, solved at atomic resolution by cryoEM [93]. Both techniques achieved similar nominal resolutions (3.5 Å for X-ray and 3.6 Å for cryoEM). At this level of detail, it is possible to observe side chains. Therefore, the polypeptide chains can be traced in the different regions of the density map with high precision. Interestingly, for AdV, tracing in the cryoEM density map proved more informative than in the X-ray diffraction density map, even at similar levels of resolution. This section summarizes the technical developments that resulted in final success for both techniques.

### 2.1. X-Ray Diffraction

The key requirement to solve a structure by X-ray crystallography is obtaining well-ordered 3D crystals of the specimen. Adenovirus, a macromolecular assemblage of 150 MDa, and ~950 Å diameter from vertex to vertex, is the largest macromolecule for which 3D crystals diffracting to atomic resolution 2 have been obtained [94]. Previously, the largest macromolecular assembly having yielded crystals diffracting to atomic resolution was the adenovirus-like bacteriophage PRD1 (70 MDa, 700 Å) [95,96,97].

Crystallization of biological macromolecules, and of these large viruses in particular, requires surmounting two main obstacles: first, production of large amounts of specimen at high concentration; second, the specimen needs to be highly homogenous to yield well-ordered crystals. This includes the requirement for stability over the crystallization and data collecting procedures (weeks), and the removal of flexible regions. The most prominent flexible regions in AdV are the long protruding fibers, particularly in human AdV species C (~300 Å), the one most used for vector and structural studies. A HAdV-5 fiber-deleted vector was initially used for crystallization trials. High concentrations (5–10 mg/mL) and crystals were obtained, but they did not yield useful diffraction data. Lack of order in these crystals may have arisen from heterogeneity of the fiberless viruses, as these seem to suffer from DNA leakage, presumably by unstable penton base incorporation in the absence of fiber [55].

Better results were obtained with a HAdV-5 chimera where the fiber was substituted by that of species B HAdV-35, approximately 1/3^rd^ the length of the HAdV-5 fiber (Ad35F) [43]. The combination of the shorter fiber, viral production in large-scale cell culture devices, and robotic crystal screening, finally produced the long sought diffraction quality crystals [94]. These were grown from a 12 mg/mL solution (~5 × 10^13^ viral particles/ml), and reached sizes in the 0.2 mm range. However, structure solution was far from straightforward, as data had to be collected from hundreds of crystals, and only about 10% were usable for obtaining a high resolution map [3]. Phasing was facilitated by the availability of a cryoEM derived quasi-atomic model [43], from which initial phases could be derived. However, electron density corresponding to minor coat proteins was hard to interpret, due to unclear connectivity and lack of significant side chain densities that could guide polypeptide chain tracing. Nevertheless, it was possible to unequivocally assign the location of polypeptide VIII; to discern the disposition of mobile regions that could not be observed in the structure of isolated hexon; and to detect an intriguing change in penton conformation. All these points will be discussed in detail in Section 3.

### 2.2. Cryo-EM

In the past two decades, cryoEM followed by image analysis techniques has steadily progressed from providing low-resolution molecular envelopes to genuine atomic resolution maps [98]. The bases of this progress and possible future developments have recently been reviewed [93,99]. High quality raw data are now obtained thanks to instrumental advances such as high beam coherence provided by field emission electron sources; more stable cryo-sample holders; and better electron lenses and microscope alignment. On the other hand, computing advances allow for image processing procedures dealing with much larger number of images, sampled at finer pixel sizes, and with more precise aberration compensation, alignment and reconstruction algorithms. For AdV, atomic (3.6 Å) resolution was achieved using a final dataset of 31,815 HAdV-5 particles, sampled at 1.076 Å/px [4]. As in the case of the crystallographic analysis, map interpretation was aided by previous knowledge coming from hybrid X-ray-cryoEM approaches. However in this case, the map quality was such that polypeptide chains could be traced using some distinctive, bulky side chain densities as landmarks. The cryoEM analysis revealed in detail the structure of minor coat proteins, and how they contribute, together with mobile arms in hexon and penton base, to establish the complex network of interactions involved in organization of the icosahedral protein shell. This is described in detail in the next section.

## 3. Atomic Structure of the Mature Adenovirus Virion

**Figure 2:**
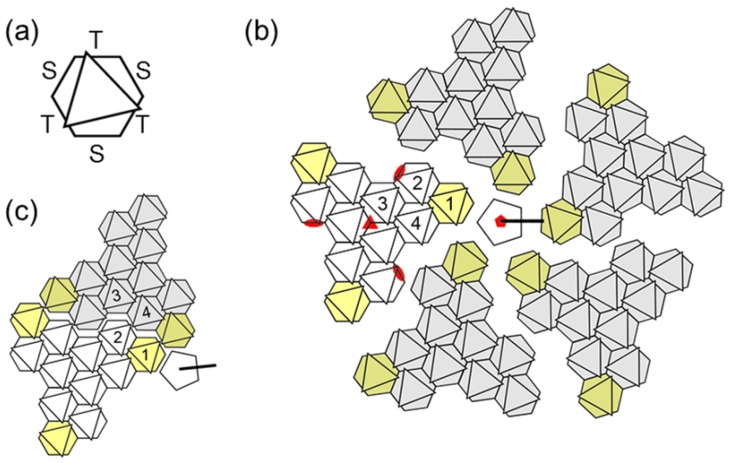
**Geometrical landmarks in the AdV icosahedral net.** (**a**) Each hexon trimer is depicted as a hexagon with an overlaid triangle indicating the position of the towers. The two different kinds of faces in the hexon pseudo-hexagonal base are labeled S (single monomer) and T (two monomers). (**b**) One penton and the five adjacent facets are represented, spread out for clarity. The four hexon trimers forming the asymmetric unit (AU) are numbered in one facet. Peripentonal hexons, forming the Group of Six (GOS) together with the penton, are highlighted in yellow. Hexons in the Groups of Nine (GON) are depicted in white for the reference facet, gray in the rest. Red symbols indicate the icosahedral symmetry axes. Notice that there is an alternative way to define the AU, in which hexons belonging to two facets would be included (**c**). This alternative AU was used in the atomic resolution cryoEM study.

To describe the complex AdV structure, it is useful to depict the capsid using a series of geometrical landmarks. Each capsid facet is formed by 12 trimers of the major coat protein, hexon. As explained above, hexon trimers have a hexagonal shape, and so they can act as hexagonal bricks in the icosahedral network. The pentagonal elements at each vertex are formed by a pentamer of penton base, in a complex with a trimer of the long projecting fiber. Four hexon trimers, plus one penton base monomer, form the icosahedral AU. The general icosahedral architecture can be described as two different systems of tiles. Nine hexon trimers form the central plate of each facet or GON. Similarly, the five peripentonal hexon trimers, together with the penton base, can be considered as a second tile system, which has been designed as GOS (Group of Six) [4] (Figure 2).

### 3.1. Hexon

Hexon is the main building block of the AdV capsid (copy number = 720 monomers, 240 trimers). The pseudo-hexagonal symmetry of the hexon trimer base provides two kinds of hexon-hexon contact faces. One is composed by the two β-barrels belonging to the same monomer, while the other is formed by two barrels belonging to two different monomers. Before the atomic structure of hexon was solved, these faces were denoted as type B and A, respectively [33]. This nomenclature has also been used to describe other members of the structural lineage, such as PRD1 [100]. In the atomic resolution cryoEM model, these are called S (for Single monomer), and T (Two monomers). Intrafacet interactions are of the ST type, while SS and TT interactions make up the icosahedral edges. Additionally, the peripentonal hexon interacts with penton base by an S face (Figure 2).

The intricate folding and remarkable stability of the hexon trimer has been extensively described [35,61,101,102,103,104]. The atomic resolution maps of the complete virion show no changes in hexon conformation with respect to the crystallographic structures of the isolated protein. However, they do show the structure of some regions that due to their flexibility could not previously be traced at high resolution. The role of the N- and C- termini of the hexon monomer is now unveiled. Depending on their context in the capsid, these regions, located at the innermost part of the hexon base, adopt different conformations to establish interactions between hexons, or between hexons and minor coat proteins IIIa and VIII. This is a common theme in viral coat proteins: often they display mobile terminal regions in solution, which serve as conformational switches modulating the different quasi-equivalent interactions within the icosahedral capsid [96,105,106,107,108].

Mobile regions could also be traced on the outermost part of hexon. In the crystallographic study [3], two mobile loops in the hexon towers were observed thanks to their stabilization by crystal packing interactions. In the cryoEM structure, four of these hexon loops were traced. Some of these correspond to hypervariable regions 4 to 7, involved in defining the specific serological response to the virus [4]. Interestingly, in recent years a function for hexon beyond the purely architectural one has been unveiled, also involving the hypervariable regions. In two parallel studies, it was shown that coagulation factor X, critical for mediating AdV infection of hepatic cells *in vivo*, strongly bound hexon in the central hollow region between the towers, lined by hypervariable regions 3 and 7 [58,59]. This unexpected finding confers to hexon a role in tropism determination, receptor binding and entry; all these aspects had previously been reserved to the penton proteins.

### 3.2. Penton Base and Fiber

Penton base (polypeptide III) and fiber (polypeptide IV) form the vertex capsomers, key players in the initial stages of infection. The distal, C-terminal fiber knob is responsible for initial attachment to the host cell by binding to the cell surface protein CAR [109] in most human serotypes. Fiber flexibility is important for this interaction to take place [53], presumably to allow the fiber to bend away. Then, an RGD loop in penton base binds to α_V_ integrins to trigger internalization by receptor-mediated endocytosis [110]. Tracking of fluorescent single viruses or fiber knobs upon attachment has recently revealed how CAR-related drifting and integrin-related confinement combine to start viral uncoating, and therefore productive infection [111].

**Figure 3:**
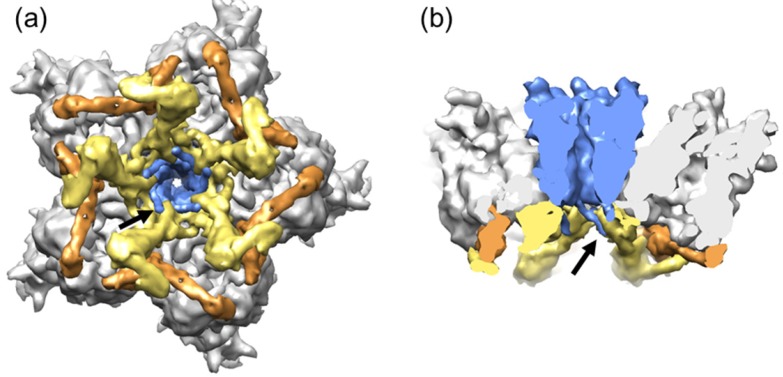
**Organization of the AdV vertex region (GOS).** (**a**) View from inside the capsid looking at the 5-fold icosahedral symmetry axis. (**b**) Side view showing a transversal section across the penton. The five peripentonal hexons are shown in gray; penton base in blue; polypeptide IIIa in yellow; and the GOS copy of polypeptide VIII in tan. Surfaces were created from high resolution structures in PDB ID 3IYN [4], using UCSF Chimera software [113]. Notice the N-terminal penton base arms (arrows) intercalating between IIIa monomers, and the radial position of polypeptide VIII, wedged between IIIa and hexon.

The penton base structure in the cryoEM map of the complete virion [4] is almost indistinguishable of that reported for the HAdV-2 recombinant protein in the presence of an N-terminal fiber peptide [42]. Similarly to hexon, the only differences reside in the visibility of flexible, previously untraced areas. For each penton base monomer, an N-terminal arm (residues 37–51) extends away from the β-barrels that form the main body of the protein towards the viral core, interacting with the N-terminal domains of two IIIa monomers along the way, and therefore contributing to anchor the penton within the GOS (Figure 3). The relevance of this interaction is reflected in the observation that incorporation of exogenous peptides at the N-terminus of IIIa, even when allowing assembly, resulted in virions with a tendency to lose pentons [112].

Fiber density is usually absent or blurred in structural studies of the complete virion, due to its intrinsic flexibility and the symmetry mismatch between the trimeric fiber and the 5-fold icosahedral vertex. In the crystal structure of recombinant penton bound to the N-terminal fiber peptide, residues 10–20 were observed bound at the cleft formed by two adjacent penton base monomers [42]. In the cryoEM atomic structure, fiber residues 7–19 could be traced, and density for the proximal part of the fiber shaft was observed, extending the information obtained from the previous studies [114]. The base of the fiber interacts with a hydrophobic ring at the rim of a narrow channel in the center of the penton base pentamer. Interestingly, the N-terminal fiber peptide reaches from the central shaft to the RGD loop in the penton base periphery, consistently with previous studies reporting conformational changes in the RGD loop upon fiber binding [115].

Unlike in the cryoEM structure, in the crystal structure of the complete virion an intriguing conformational alteration of penton base was found [3]. The pentamer subunits appear arranged around a channel with almost double the diameter observed for the isolated protein (50 *vs.* 28 Å). This implies that the fiber shaft could be inserted inside the penton base, instead of being attached to the surface as indicated by the recombinant protein study and the cryoEM structure of the complete virion [42,114]. There are two possible reasons for this remarkable change in the penton conformation. First, for the crystallographic studies a pseudotyped virus was used, with the long and flexible HAdV-5 fiber substituted by the short HAdV-35 one. The different penton conformation would then suggest a different mode of fiber-penton base binding for serotypes with short, rigid fibers. Another possibility is that a transient penton conformation has been selected by the crystallization conditions or the constraints imposed by crystal contacts. Indeed, some plasticity in the penton base architecture is likely to be involved in its role during infection, from sealing the packed genome to triggering stepwise dismantling upon receptor binding. Structural changes in penton base have been reported both upon fiber and integrin binding [52,115].

### 3.3. Polypeptide IIIa

There is one monomer of IIIa per AU of the icosahedral particle, with a total copy number of 60. Five IIIa monomers are arranged in a ring underneath each vertex, at the inner capsid surface [4,44,112] (Figure 3). Most residues from 7 to 300 (out of 585) could be traced in the cryoEM map, forming three domains with predominantly helical fold. The N-terminal domain of polypeptide IIIa keeps each GOS together by tethering pairs of peripentonal hexons, and those to penton base. It was therefore termed “GOS-glue domain”. This is linked by a long helix to another region (“VIII-binding domain”) interacting with one of two independent copies of polypeptide VIII. This interaction tethers the GOS to the hexons in the central plate of the facet (GON). The remainder of the polypeptide IIIa chain (residues 300 to 585) was not observed, implying that it does not follow icosahedral symmetry. This C-terminal region may be interacting with the non-icosahedral core.

Based on the effect of mutations or extensions of IIIa in capsid assembly [112,116], and by comparison with the corresponding minor coat protein in bacteriophage PRD1 [96], two possible roles had been proposed for IIIa in the viral cycle: stabilizing the vertex region and the packaged genome upon assembly, and signaling for vertex and genome release during uncoating. The cryoEM structure of the complete virion supports the role of IIIa in stabilization of the vertices. More recently, it has been shown that serotype specific interactions between the N-terminal domain of IIIa and the putative scaffold protein L1 52–55K are required to promote correct genome packaging [117].

### 3.4. Polypeptide VI

**Figure 4:**
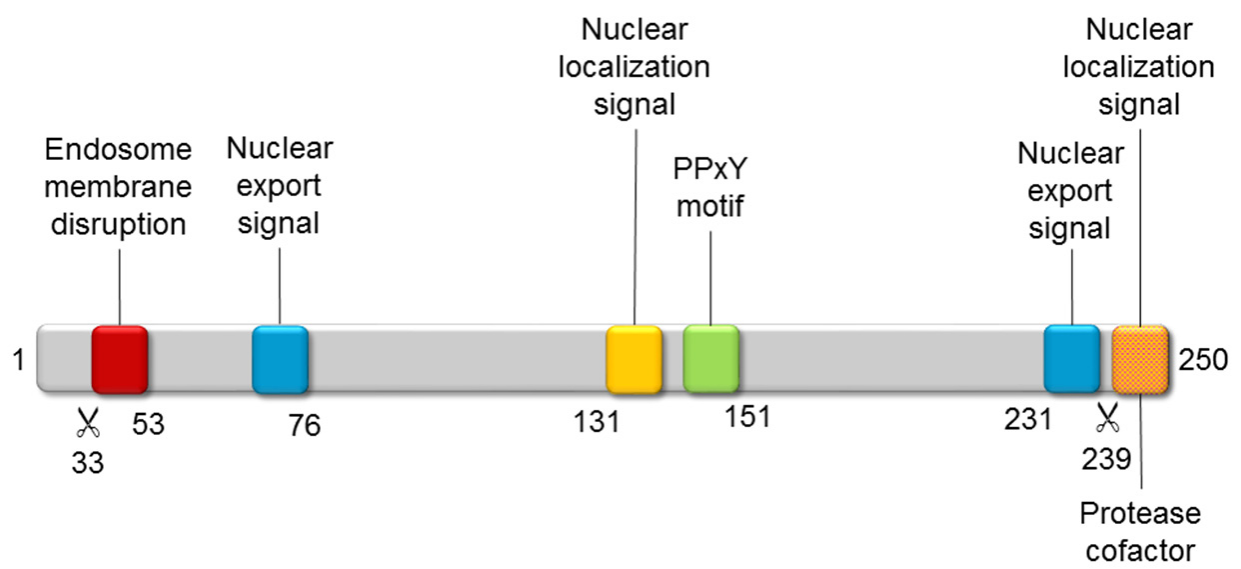
**Functional motifs of polypeptide VI.** The adenoviral protease (AVP) cleavage sites (scissor symbol) and the position of some residues are indicated below the gray bar representing the precursor polypeptide VI polypeptide chain.

Polypeptide VI is a remarkable, multifunctional protein playing multiple roles throughout adenovirus infection (Figure 4). During entry, its N-terminal amphipathic helix alters the curvature of the endosomal membrane, so that the virus can escape into the cytosol [118,119,120]. A ubiquitin ligase interacting motif (PPxY) has a role in facilitating trafficking to the nucleus along the microtubular network [121]. Moreover, the PPxY motif is also required for VI to act as an activator of the adenoviral gene expression [122]. Later on, a nuclear localization signal located at the C-terminus of VI interacts with importin α/β, to promote transport of newly synthesized hexon bound to VI to the nucleus [123]. Finally, this same C-terminal peptide is substrate and cofactor of the adenoviral protease (AVP), facilitating its action on multiple structural proteins to yield the infectious viral particle (see Section 4) [124,125].

Polypeptide VI binds to a loop in the inner cavity of the hexon trimer, and to dsDNA [123,126]. These interactions place it inside the capsid, bridging the core to the icosahedral shell. Both cryoEM and crystallographic studies find weak, fragmented density within, or close to, the hexon cavity, that could correspond to part of polypeptide VI [3,4,44]. However the density quality was not enough to assign it to any particular sequence. The copy number of VI, estimated around 360, is too low to have one molecule of VI per hexon monomer (copy number 720), and too high to have one VI per hexon trimer (copy number 240). Density weakness indicates that the arrangement of VI within the hexon cavities of the mature virion does not follow icosahedral symmetry, and is therefore blurred in the structural studies where icosahedral symmetry is explicit or implicitly enforced.

### 3.5. Polypeptide VIII

Of all the AdV minor coat proteins, polypeptide VIII is probably the least characterized regarding its function. A mutation mapped in the VIII gene has been reported to result in a thermolabile phenotype, which would be in agreement with its architectural contribution to the capsid [127]. On the other hand, porcine adenovirus type 3 (PAdV-3) polypeptide VIII has been shown to interact with the putative packaging motor IVa2, hinting at some kind of role in genome packaging [128]. Locating polypeptide VIII in the virion structure was also difficult, and it was only when the cryoEM studies reached 10 Å resolution that this minor coat protein could be assigned to certain internal capsid densities [45]. These positions are now confirmed by both the cryoEM and the crystallographic atomic structures [3,4].

**Figure 5:**
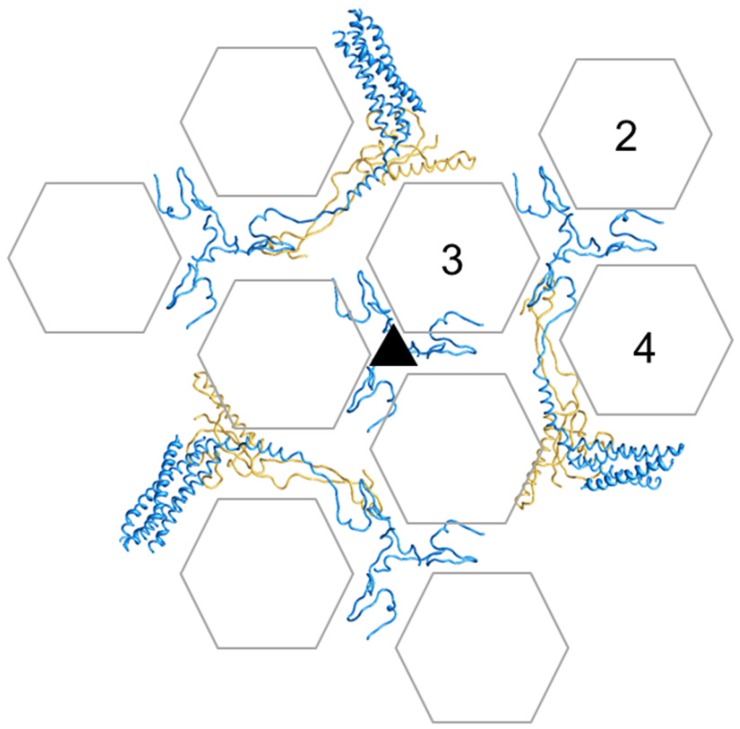
**Cementing of the GON.** Organization of minor coat proteins VIII and IX in the GON. The four copies of IX in one GON are shown in blue, and the three copies of VIII in yellow. Only the fragments of IX that could be traced in the atomic resolution cryoEM map are depicted. Hexon positions are represented as transparent hexagons to allow simultaneous view of IX and VIII. Hexons in one AU are labeled 2 to 4 (hexon 1 is not a part of the GON). The view is from outside the capsid.

There are two independent monomers of polypeptide VIII in the AU. One of them is wedged between the polypeptide IIIa VIII-interacting domain and the hexon bases at the periphery of the GOS, cooperating with IIIa to bind each GOS to its five surrounding GONs (Figure 3). The second copy is located around the icosahedral 3-fold symmetry axis, contributing with polypeptide IX (see next section) to the stabilization of each GON (Figure 5). Although early studies suggested that polypeptide VIII was part of the GON, later reports consistently found only hexon and polypeptide IX, indicating that the interaction of VIII with hexons may be less stable than that of IX [36,37]. Each independent copy of VIII interacts with four hexon trimers, regardless of whether they belong to the GOS or the GON. Interestingly, the interactions of VIII with both polypeptide IIIa and hexons occur by β-sheet augmentation. That is, the interaction is mediated by a β-strand from polypeptide VIII binding to the edge of a β-sheet in the other protein [129].

**Figure 6:**
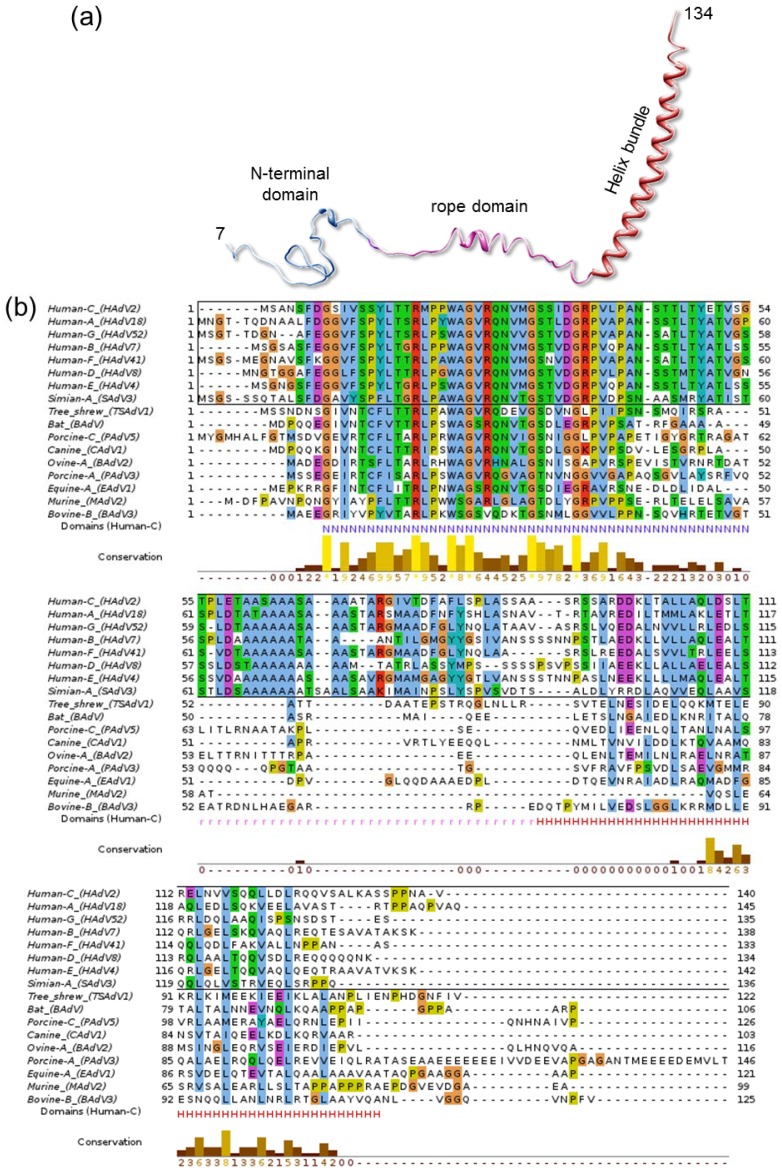
**Polypeptide IX structure and conservation.** (**a**) The extended fold of polypeptide IX is shown as a ribbon, with the different domains labeled and depicted in different colors. (**b**) Alignment of representative polypeptide IX sequences for the different species in the *Mastadenovirus* genus. In the bottom line the extent of the N-terminal (N), rope (r) and helix bundle (H) domains from the HAdV-5 structure are indicated with letters in the same color used for each domain in (a). Notice that the rope domain is conserved only in human and simian viruses (sequences within a black rectangle). Sequences were retrieved from UniProt [143] and aligned with Clustal [144] and JalView [145]. The extra-long PAdV-3 IX sequence is shown truncated for figure clarity.

The cryoEM study also shed light into the fate of the different proteolytic maturation products of polypeptide VIII. Polypeptide VIII is synthesized in precursor form (pVIII, 227 residues), present in assembly intermediates [130,131]. It has three potential cleavage sites by AVP at residues 112, 131 and 157 [132], but it was not clear which ones were actually cleaved, nor which fragments remained in the virion (Figure 6a). While some mass spectrometry studies indicated that both an N- and a C- terminal fragment were present in mature virions [133,134,135], others detected only the N-terminal [136] or the C-terminal [130] fragments. In the cryoEM map, practically all residues in the pVIII sequence could be traced, except those between Gly110 and Arg159 [4]. This confirms that both larger fragments (2–112 and 157–227) stay in the virion. Further, they stay together and in an ordered form, presumably conserving the fold adopted by the precursor pVIII. Lack of density for the 45 central residues indicates that they are either removed, or become disordered upon proteolytic processing. At least residues 132 to 157 remain in the virion as a unity, since this peptide has been detected by mass spectrometry [134]. In PAdV-3, both the precursor form pVIII, as well as the two largest fragments pVIII_N_ and pVIII_C_ were observed to interact with packaging protein IVa2 [128]. 

### 3.6. Polypeptide IX

Polypeptide IX is the only minor coat protein located on the outer part of the AdV capsid. The cryoEM atomic structure [4] revealed how polypeptide IX forms a sort of hairnet on the outer side of the virion, keeping together the hexon trimers in each GON and binding GONs to GONs across the icosahedral edges. The 140 residue polypeptide has an extended and remarkably flexible conformation that extends as far as 185 Å, reaching all the way from the GON 3-fold axes to the protruding helical bundles at the facet edges (Figure 5 and Figure 6a). The N-terminal domains of three IX monomers join via hydrophobic interactions at the icosahedral and local 3-fold axes in the GONs forming the triskelion structures that had already been observed in the first difference imaging studies of GONs [38], and interacting with hexons via yet another β-strand augmentation. Then the so-called “rope domain” of each monomer runs in a different direction towards the facet edges, where the C-terminal α-helix joins with the C-terminal helices of another three copies of IX, different from those forming the N-terminal triskelion, to create a leucine zipper 4-helix bundle. Remarkably, one of the helices in the bundle belongs to a monomer of IX from the neighboring facet, and runs antiparallel to the other three. Thus, 12 monomers of IX form four triskelions, but only three helical bundles per icosahedral facet. Consistent with this intricate interlacing, incorporation of polypeptide IX to the capsid seems to be highly cooperative, so that virions either have the whole IX complement or no IX at all [46]. Also, the 4-helix bundle appears to be disorganized by C-terminal fusion of exogenous peptides or by antibody binding; however this does not preclude incorporation of IX to the capsid [47,48]. One of the copies of IX in the facet follows a path parallel to the GON copy of VIII in the inner capsid surface. The more structured body domain of the VIII copy in the GON is directly below a IX helix bundle; then the neck domain of VIII runs parallel to the rope domain of IX, to finish close to the triskelion located at the local 3-fold axis (Figure 5). Interestingly, this copy of IX that runs parallel to VIII was better ordered than the rest and was the only one allowing tracing of the complete polypeptide chain.

It was long known that IX was dispensable for assembly but had a capsid stabilizing role, as IX-deletion mutants assembled viral particles but presented lower thermostability [137]. The trimeric N-terminal domain of IX is enough to confer capsid thermostability [138]. Its stabilizing role and organization in the capsid bring to mind other accessory proteins such as *Soc* in bacteriophage T4, which also trimerizes to form a network of clamps between neighboring capsomers [139]. A difference between these proteins is that *Soc* is incorporated into the capsid in the latest maturation stages [140], while AdV polypeptide IX is already present in assembly intermediates (empty or immature virions). An additional *in vivo* biological function for polypeptide IX has recently been proposed [141]. A IX-deleted virus was observed to efficiently transduce CAR-negative cell lines, have enhanced monocyte activation capacity, and change the *in vivo* distribution in mice. Therefore, IX may play a role in modulating the viral tropism and/or interfering with the immune response.

Indeed, some other properties of IX hint towards a possible role in interaction with the host. Of all characterized AdV genera, polypeptide IX is present only in *Mastadenovirus*, comprising only mammalian-infecting species. Other genus-specific polypeptides play a similar architectural role in the capsid, such as protein LH3 in the broad host range *Atadenovirus* genus [50]. Even within the *Mastadenoviruses*, polypeptide IX considerably varies from host to host. In particular, the central region defined in the cryoEM structure of HAdV-5 as the rope domain (Figure 6a) is conserved only among human and simian AdVs (Figure 6b), while for example canine virus CAdV-1 has a shorter IX protein lacking this domain. This results in the helical bundle being located directly on top of the triskelions, instead of at a distance at the facet edges like in HAdV [51]. In any case, all IX and IX equivalent polypeptides observed in structural studies so far have at least one domain clearly exposed on the capsid, in an appropriate location to interact with host partners. This exposed position has made it a popular platform for adenoviral vector modification for retargeting, imaging or immunization [142].

Finally, a role for IX during viral entry has recently been reported [146]. After escaping from the endosome, the partially disrupted virion travels along the microtubule network towards the nuclear pore [147], where final disassembly takes place to promote transport of the viral genome into the nucleus. A complex set of interactions ensues whereby capsids are anchored to the NPC while simultaneously being pulled away by microtubule motor kinesin-1, resulting in capsid dismantling and fragment release into the cytosol. Immunoprecipitation assays indicated that polypeptide IX was the virion component responsible for interaction with kinesin-1 via its light chain Klc1/2.

## 4. Structural Changes Involved in Maturation

**Figure 7:**
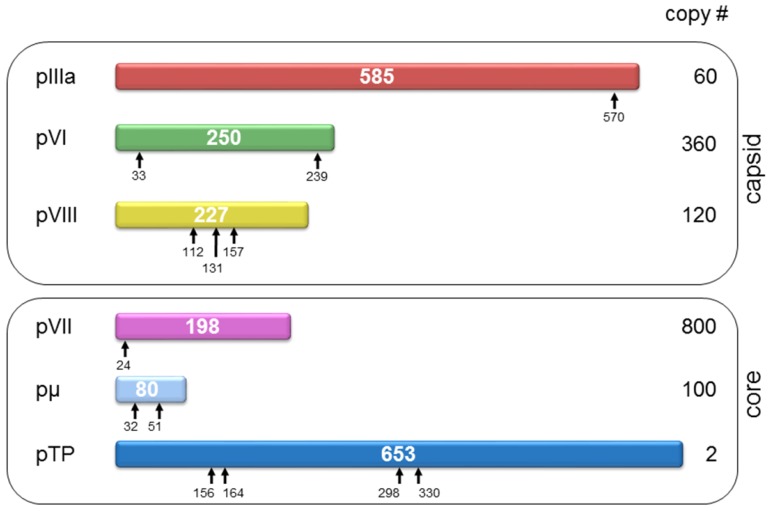
**Virion components undergoing proteolytic maturation**. Each HAdV-5 precursor protein is represented as a bar with the polypeptide length in amino acids indicated in the center. Cleavage sites are denoted by arrows. There are four potential cleavage sites in pTP but they have not been experimentally verified. The prefix “p” denotes the unprocessed precursors.

As described above, the structure of AdV in its final, infective forms has been extensively studied. However, the structural aspects of the pathway leading to this final form are less well understood. Only recently cryoEM has yielded structural information of some assembly intermediates: namely, the immature (also called young) virion [148]. Like many other viruses, AdV needs to undergo a number of proteolytic cleavages to become infective. The agent responsible for proteolytic maturation is the viral L3 23K protein, or AVP [149]. AVP recognizes (M/I/L)XGX-G and (M/I/L)XGG-X sequence motifs to cleave minor capsid proteins IIIa, VI and VIII, as well as core proteins VII, μ, and the terminal protein (TP) [132] (Figure 7). Without these cleavages, the immature particle lacks infectivity because of its inability to uncoat [150,151].

One striking particularity of the adenoviral protease, which differentiates it from all other viral proteases described so far, is its use of the viral DNA as a cofactor. Indeed, the AVP enzymatic activity is increased 100 fold in the presence of DNA. Another cofactor is the C-terminal peptide of precursor polypeptide pVI (pVI_C_), released upon cleavage by AVP. Together, these cofactors increase the protease catalytic rate by several orders of magnitude [124,125,152,153]. It has been proposed that AVP uses the dsDNA molecule as a 1-dimensional track to diffuse in the crowded capsid environment and reach all its target substrates [153].

A HAdV-2 thermosensitive mutant (*ts1*) is a useful experimental system to investigate the structural and functional aspects of maturation [154]. When grown at the non-permissive temperature (39 °C), *ts1* does not package AVP [155], and produces capsids containing the unprocessed protein precursors. Viral genome packaging is unimpaired, but the virus is not infectious. The *ts1* mutation consists of a single replacement of Proline 137 by Leucine in the AVP sequence [155]. The phenotype of this classic mutant has now been reproduced by genetical engineering in both HAdV-5 and HAdV-2 [156,157].

**Figure 8:**
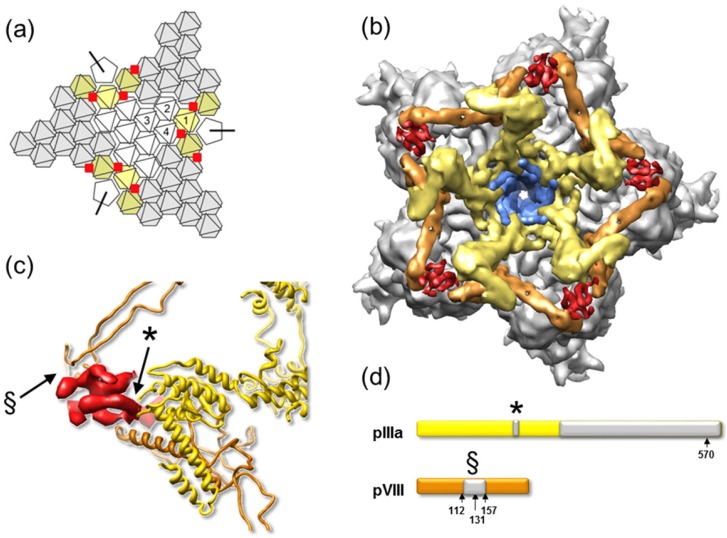
**The molecular stitch in immature AdV.** (**a**) Schematics showing four adjacent icosahedral facets, with the position for the molecular stitch inside the capsid in red. (**b**) The GOS represented as in Figure 5, with the density for the molecular stitch derived from the *ts1*-WT difference map at 8.9 Å in red. The five peripentonal hexons are shown in gray; penton base in blue; polypeptide IIIa in yellow; and the GOS copy of polypeptide VIII in tan. View from inside the capsid looking at the 5-fold icosahedral symmetry axis. (**c**) Zoom into the region close to one of the molecular stitches with the structures of IIIa and VIII from [4] represented in ribbons. (**d**) Bars representing the precursor polypeptides IIIa and VIII with the cleavage sites indicated (arrows). Polypeptide regions not traced in the cryoEM atomic structure are in gray. Untraced regions close to the molecular stitch are indicated with the same symbol in (c) and (d).

Two cryoEM studies have analyzed the structural differences between AdV mature and immature capsids by comparison of wild type (WT) and *ts1* structures obtained at 10 and 8.5 Å resolution [1,2]. These studies indicated three main differences between the mature and immature virions. First, in the inner capsid surface of *ts1* there are extra densities located between the ring of peripentonal hexons and those making the GON. The authors called these densities a “molecular stitch”, a structure that would contribute to hold the GOS in place during assembly, but needs to be removed afterwards to facilitate vertex release for uncoating [158]. With the data available at the time, this feature was assigned to the C-terminal peptide of pIIIa. In the light of the atomic structure published later [4], the identity of this “stitch” becomes more clear (Figure 8). Indeed, this feature is in close proximity to two regions where polypeptide chains no longer could be traced in the atomic resolution map, either because of their absence or because of disorder. These regions are: a short stretch of residues in polypeptide IIIa VIII-binding domain (residues 216–225); and the 45 residues between the cleavage sites at residues 110 and 159 in polypeptide VIII (see Section 3.5). The extra density was only observed close to the peripentonal copy of VIII, and not in the second independent copy underneath the GON. Therefore, it seems likely that the molecular stitch is a structure formed by the contribution of the central peptides of uncleaved pVIII and IIIa, adding an additional element to the complex network of interactions holding the capsid together.

The second difference observed was another extra density located inside all hexon cavities in the *ts1* structure [1,2]. Weak density has been observed at this location in all sub-nanometer resolution structural studies of the mature particle, and has been attributed to polypeptide VI [3,4,44] (Section 3.5). Stronger density in 3DEM maps of *ts1* therefore indicates that when the precursor form pVI is present, the interaction with hexon is different, resulting in a more uniform occupancy or ordering of the part of VI inserted within the internal hexon cavity. This interaction change relates to the different functions of VI during the viral cycle: a stronger bond to hexon would be required for the transport function in assembly, while a looser interaction would facilitate release of VI from the capsid in the endosome.

Finally, the third difference observed between mature and immature viruses concerned the core organization. This is remarkable in itself, due to the scarce data available on the disposition of DNA and accompanying proteins within the virion, and because it confers the core architecture a role in infectivity. Although some details differed, both cryoEM studies indicated that the core undergoes a transition from a more ordered to a more disorganized structure during maturation [1,2]. Disrupted *ts1* virions released compact, spherical cores, hinting at an extra stabilization of the structure. This suggests that precursor proteins pVII and pµ have a much stronger dsDNA condensing activity than their mature versions. The immature core exhibits increased thermostability, and unravels forming a 12 nm thick nucleoprotein filament that exits the capsid trough a single vertex, bringing to mind the unidirectional genome packaging and possible existence of a singular vertex [2,28,159].

## 5. Remaining Questions

AdV has long been a subject of interest for structural biology, both because of its intrinsic biological relevance and because of the challenges it poses. Work by many researchers culminated in 2010 with the resolution of the atomic structure of the virion by cryoEM and crystallography. However, plenty of questions are still open to understand the structural determinants of the AdV infectious cycle. A large part of the success in structural studies comes from taking advantage of the 60-fold averaging facilitated by the icosahedral architecture. However, this same advantage may turn into a disadvantage, since information on the non-icosahedrally ordered viral components is blurred and therefore lost in the averaging process. These components, however, may play critical roles in the viral cycle. Such is the case of the viral genome, and in the case of AdV, its large entourage of DNA-bound proteins. There are no structural data for any of the core proteins (polypeptides V, VII, μ), as well as for a large part of the polypeptide IIIa chain (residues 301 to 585). It is not known how the 12 μm long molecule of dsDNA, plus over 25 MDa of protein, fit into the ~0.1 μm diameter capsid. Unlike for its structural partner bacteriophage PRD1, AdV 3DEM maps do not show a concentric ring pattern inside the capsid [100]. Further, ordering of the nucleoproteic core changes during viral maturation [1,2]. 

It is still not clear how AdV capsid size is determined. Theoretical studies based on the general shape of hexon, and later structural studies, have shown that capsids with different sizes and triangulation numbers can be made using the same kind of hexagonal shaped trimeric capsomers [33,88]. In bacteriophage PRD1, an extended minor coat protein runs beneath the icosahedral edges and has been proposed to act as a tape measure for capsid building [96]. In AdV, both polypeptide VIII and IX have been proposed to act as molecular rulers [4,96]. However, VIII does not have the extended conformation and edge location expected for such a protein; and capsids with the correct size can be assembled in the absence of IX [45,46,137]. Nor are the putative packaging motor IVa2 or the core components responsible for size determination, as empty capsids with uniform size are made in the absence of IVa2 and/or packaged genome [160].

Finally, another area where considerable unknowns remain is the temporal pathway of assembly. Is capsid assembly and DNA packaging sequential, or is it a simultaneous process? How would a protein-covered dsDNA molecule be translocated through a portal-type packaging complex? How about proteolytic maturation? Does it occur after the capsid is full and sealed, or is it concomitant with packaging? How could the compact state of the immature core be reconciled with packaging into a preformed capsid? It can be expected that the latest advances in structural studies reviewed here form a firm basis to build on towards elucidation of these intriguing questions.

## Footnotes

^1^ HAdV: human adenovirus. FAdV: fowl adenovirus. CAdV: canine adenovirus. SAdV: simian adenovirus. PAdV: porcine adenovirus.

^2^ Strictly speaking, “atomic resolution” would be ~1 Å. However it is frequent to use this term, like here, meaning “a resolution such that the map quality allows tracing of the polypeptide chain, generally thanks to the visibility of bulky side chains”.
