# San Martín, C., Correction: Latest Insights on Adenovirus Structure and Assembly. *Viruses* 2012, *4*, 847-877

**DOI:** 10.3390/v4123952

**Published:** 2012-12-19

**Authors:** Carmen San Martín

**Affiliations:** Department of Macromolecular Structures, Centro Nacional de Biotecnología (CNB-CSIC), Darwin 3, 28049 Madrid, Spain; E-Mail: carmen@cnb.csic.es; Tel.: +34-915855450; Fax: +34-915854506

It has come to my attention that my article "Latest Insights on Adenovirus Structure and Assembly" (*Viruses* **2012**, *4*, 847-877) [1] contains an inaccurate statement. 

On page 864, the caption for Figure 7 reads: "There are four potential cleavage sites in pTP but they have not been experimentally verified". However, three of these sites have been experimentally confirmed *in vitro* using recombinant AVP and pTP, as described in Webster A, Leith I.R., Hay R.T.: Activation of adenovirus-coded protease and processing of preterminal protein. *J. Virol.* **1994**, *68*, 7292-7300 [2].
